# Supplementary material for: Associated Factors of Mycobacterium Leprae Infection among People with Leprosy in Kwale County
Source: PLoS Negl Trop Dis. 2025 Nov 25;19(11):e0012901. doi: 10.1371/journal.pntd.0012901 (PMC12677770; doi:10.1371/journal.pntd.0012901)
Supplement: S1 Table — (DOCX) [file pntd.0012901.s001.docx]

A screening Checklist Questionnaire for the controls

| YES/NO | | | | | | | | | | | |
| --- | --- | --- | --- | --- | --- | --- | --- | --- | --- | --- | --- |
| 1 | Do you have difficulty with seeing, even when wearing glasses? | | | | | | | | | |  |
| 2 | Do you have difficulty walking or climbing steps? | | | | | | | | | |  |
| 3 | Do you have difficulty washing all over or dressing? | | | | | | | | | |  |
| 4 | Are there activities that you cannot perform? | | | | | | | | | |  |
| 5 | Do you have a loss of feeling in the hands and/or feet? | | | | | | | | | |  |
| 6 | Do you have any weakness in your hands and/or feet? | | | | | | | | | |  |
| 7 | Do you have any wounds on your hands and/or feet? | | | | | | | | | |  |
| 8 | Do you have any problems in relationships or in taking part in festivities, work, meetings, etc? | | | | | | | | | |  |
| 9 | Have you ever been diagnosed with leprosy previously? | | | | | | | | | |  |
| 10 | Has any member of your family been diagnosed with leprosy? | | | | | | | | | |  |
| 11 | Has any of your friends been diagnosed with leprosy | | | | | | | | | |  |
| 12 | Presenting signs/ symptoms | | | | | | | | | |  |
| 13 | Duration of symptoms in months | | | | | | | | | |  |
| 14 | Past history (of similar symptoms and treatment | | | | | | | | | |  |
| 15 | Skin examination: Total anesthetic Patches: (No. | | | | | | | | | |  |
| 16 | Skin infiltration: Yes/No | | | | | | | | | |  |
| 17 | Skin anhidrosis: Yes/No | | | | | | | | | |  |
| Nerve examination | Ulnar | | Median | | Radial | | LP | | PT | |  |
|  | R | L | R | L | R | L | R | L | R | L |  |
| Thickened |  |  |  |  |  |  |  |  |  |  |  |
| Tender |  |  |  |  |  |  |  |  |  |  |  |
| 18 | | signs of the disease with demonstrated presence of bacilli in skin smear or histopathological confirmation | | | | | | | | | |
| 19 | | Hypo-pigmented skin lesions with loss of sensation | | | | | | | | | |
