# Supplementary material for: Associated Factors of Mycobacterium Leprae Infection among People with Leprosy in Kwale County
Source: PLoS Negl Trop Dis. 2025 Nov 25;19(11):e0012901. doi: 10.1371/journal.pntd.0012901 (PMC12677770; doi:10.1371/journal.pntd.0012901)
Supplement: S1 Text — (DOCX) [file pntd.0012901.s002.docx]

**Data Abstraction Tool**

| Social- Demographic Information | | | |
| --- | --- | --- | --- |
| Serial Number |  | Sub-County registration Number |  |
| Health Facility |  | County |  |
| Patient Name |  | | |
| Sub-County |  | Ward |  |
| Village |  | Physical Address, email address, phone number |  |
| Alternative Phone Number |  | | |
| Age |  | Sex |  |
| Marital Status |  |  |  |
| Clinical information | | | |
| BMI |  | Type of leprosy |  |
| Type of patient |  | Disability of the eye |  |
| Disability of the hand |  | Disability of the feet |  |
| Disability Grade |  | Skin smears  Results |  |
| The date started on MDT |  |  |  |
| TB Co-infection |  | HIV Status |  |
| Other Co-infection |  |  |  |
| Treatment Outcome |  |  |  |

(MOH, 2021)
