# Supplementary material for: Associated Factors of Mycobacterium Leprae Infection among People with Leprosy in Kwale County
Source: PLoS Negl Trop Dis. 2025 Nov 25;19(11):e0012901. doi: 10.1371/journal.pntd.0012901 (PMC12677770; doi:10.1371/journal.pntd.0012901)
Supplement: S2 Text — (DOCX) [file pntd.0012901.s003.docx]

Data Confidentiality Agreement

I as a principal Investigator/Research Assistant/CHP of 2023 leprosy verification exercise, understand that I may have access to confidential information about Health facilities and clients. By signing this statement, I am indicating my understanding of my responsibilities to maintain confidentiality and agree to the following confidentiality requirements:

I WILL treat all information collected for this data extraction process as confidential before, during, and after the assessment period. I will not use such information for any purposes other than for the work assigned to me during this exercise.

I WILL NOT share any information about the sampled clients and facilities with persons outside the analysis team. This information may include their HIV status.

I WILL refer all data-related questions asked of me that I am not authorized to disclose to the appropriate data analysis team leader or supervisors.

I WILL maintain all related data/material in a secured location at all times. I will also make sure that persons not involved in this exercise do not have access to the analysis material.

I WILL report the loss of any assessment data/material whether in paper or electronic format immediately to the analysis team lead, who is responsible for reporting this information to the TB Programme.

If I use a phone or tablet to enter or store collected information, I WILL keep that information in password-protected.

I will destroy any hard copies of materials that I may have generated in the course of the analysis before leaving the health facility.

I WILL NOT produce copies or back-up of datasets except as required. I WILL NOT misuse any information security privileges that I may have from working on this exercise.

I WILL ensure that the back-up datasets are also stored according to the confidentiality guidelines mentioned above. I WILL NOT use the results of the assessments in any way without prior authorization from the Ministry of Health.

If I cause a breach or become aware of a breach in confidentiality, I WILL take immediate steps to secure the sensitive information and inform my analysis team lead who will inform TB Programme/Moi University. I WILL fully comply with any other data confidentiality procedures that I am instructed to follow during this analysis. I understand that failure to comply with these rules and regulations could result in disciplinary action.

My signature below indicates I have reviewed, understand, and accept the above requirements. SIGNATURE: DATE (*dd/mm/yy*)
