# Supplementary material for: Associated Factors of Mycobacterium Leprae Infection among People with Leprosy in Kwale County
Source: PLoS Negl Trop Dis. 2025 Nov 25;19(11):e0012901. doi: 10.1371/journal.pntd.0012901 (PMC12677770; doi:10.1371/journal.pntd.0012901)
Supplement: S3 Text — (DOCX) [file pntd.0012901.s004.docx]

**Greetings!**

# Informed Consent and Assent form

## Introduction:

I am Mr/Mrs/Miss ; we are research assistants working for Moi University School of Public Health and Field Epidemiology

and Laboratory Training Programme (FELTP), Kenya. We are researching on the

Factors Associated with New Leprosy Diagnosis in Kwale County,Kenya, which is one of the prevalent neglected tropical diseases in your County. The interview may take up to 45 minutes. I will give you information and invite you to be part of this research, you are free to consult further in taking part or withdrawing from the study now, and whenever you want, there was no penalty. There may be some words that may seem unfamiliar during the interview or that you need help understanding. Please ask me to stop as we go through the information, and i will take the time to explain. If you have any questions, concerns or complaints about the study later, you can ask them for me by calling the principal researcher, Vallerian Karani, at this number (+254) 729785029).

## Purpose of the research

The significant geographical pattern variations of most new leprosy cases in Kenya over 10 years have been documented in Coast (64.4% and Western (14.4%) regions with Kwale Count contributing 20.3% for all reported leprosy cases in the Country. This may indicate the need to determine the independent risk factors associated with Mycobacterium leprae infection among leprosy cases in Kwale County, Kenya. In the last five years, all four sub-counties in Kwale County have reported at least one or more active leprosy disease, with more cases reported in Kinango and Msambweni sub-counties. The County Government of Kwale offers free, timely

diagnosis and treatment services of leprosy disease across all the Health facilities, with the primary objective being to provide health education, case management of leprosy cases, sensitize the catchment populations on the risk factors for leprosy disease, targeting community active cases search, contact tracing, increasing clinical suspicion index among health care workers with the ultimate aim of halting the trend and reducing morbidities and disabilities associated with leprosy disease.

## Confidentiality

We do our best to protect all the information about you and your name will not be written on the interview form. You will not be named in any of our reports. Only the study staff and investigators will know your answers to the questions.

**Your rights as a participant**: This research has been reviewed and approved by the Institution Research and Ethics Committee at the Moi university.

## Participant’s selection:

**Cases**: The data extraction tool (SI_A) was used to extract the data for all the leprosy cases documented in the Leprosy register including patients discharged from treatment 18 months earlier. Patients was selected from consenting participants at their residences/villages in the list generated in the TB and leprosy clinics within Kwale County. To achieve the sample size of 295. The Selected cases and control for each subject was matched according to sex, age (+/-10 years) and place of residence. The controls was drawn from the same villages (neighbours) as the cases and controls are interviewed in their households. From previous records over 10 years, an average of 30 -35 cases of leprosy disease are reported annually in kwale County. The cases are distributed across all the diagnostic and treatment facilities in the County. To achieve the sample size of 65 cases, we will enroll all leprosy cases including those previously treated and discharged from treatment 18 months.Upon consenting of the Cases and controls a structured questionnaire was administered.

**Controls in the village:** A comprehensive list of all households in the village or residence was obtained from the village elder. Each household was assigned a unique number and simple random sampling technique was used to select a neighboring household to cases using random numbers generated from MS excel tables. In this regard, the first household was selected systematically where, an eligible control matching the case age group category ± 10 years and sex was selected. At home, both written and oral consent/assent was sought to conduct interviews in the households irrespective of the age. If more than one eligible person was present one was randomly selected using secret balloting method by use of papers marked yes or no. In the event of absence or refusal, the household was dropped, and the next household on the randomized list was selected. This was repeated until each village had the required target of controls.

**Controls at the Household level**: At the household level, permission was requested to conduct interviews, irrespective of age. A list of individuals living in that household was developed, the individuals in the age category of the Case was identified, and if they are more than one, one was randomly selected through secret balloting by the use of papers marked yes or no. If there is no person on the homestead, the household was dropped, and the next household was selected.

Consenting individuals was asked screening questions using a checklist (Appendix 5) to ensure they meet the inclusion criteria, and later the questionnaire was administered. In case of refusal of consent or absence of household occupants, the following household was selected. This was done until each village's required controls are attained.

**Risks and benefits:** There was no known risks or benefits to you as a person participating in this study research. However, the overall impact on your community may be significant because the risks associated with leprosy disease, among other tested objectives, may be crucial in addressing the health problems associated with this infection.

Confidentiality: All information you will provide was treated with utmost confidentiality.

**Consent**: You are free to withdraw from the study now and whenever you want, there was no penalty.

**Whom to contact:** If you ever have questions about this study, you should contact the principal investigator: Vallerian Karani, Box 45, Busia, MOH-FELTP Kenya, Mobile number (254)729785029.

**Questions about your rights as a participant**, you may contact/call Proffessor.Lameck Diero Mobile number +2547914883519 and Dr. Ahmed Abaade+254722818237 who are the supervisors of this study.

Signature: ………………………………………………………... Do you agree?

Participant agrees Participant disagrees

By signing this form, "I authorize the use of my records, any observations, and findings found during this study for education, publication, and/or presentation."

I voluntarily agree to participate in this research program

□ Yes □ No

**Questions:** If you have any questions, concerns or complaints about the study, please

call **Vallerian Karani-PI** at (+254) 729785029,**Co-investigators:Proffessor Lameck Diero**- Moi University and **Dr.Ahmed Abade**-FELTP Resident Advisor who are the Supervisors for the study.

**Signatures:** Your signature below indicates that you agree to participate in this study. You will receive a copy of this signed document if you want.

Signature of participant or Guardian Date

Signature of interviewer Date

Signature of Principal investigator Date

# Assent form (Minors)

**Factors Associated with *Mycobacterium Leprae* Infection among Leprosy Cases, Kwale County.**

My name is Vallerian Karani. I am a student at Moi University and i am currently pursuing a master's degree. I am inviting you to participate in a research study about **" Factors Associated with New Leprosy Diagnosis in Kwale,Kenya*.*”**

Your caregiver knows about this study and gave permission for you to be involved. If you agree, I will ask you some questions about yourself and information related to leprosy among children. These questions will help us to understand why we have Mycobacterium infection among children in this County. This process will take about 45 minutes or less if you agree to be part of the study. You do not have to be in this study. No one will penalize you if you decide not to participate in this study. Even if you start the study, you can stop later if you want. You may ask questions about the study at any time. If you decide to be in the study, I will not tell anyone else how you respond or act as part of the study. Even if your parents or teachers ask, I will not tell them about what you say or do in the study.

Signing here means that you have read this form or had it read to you and you are willing to be in this study.

Name of the Participant (Write your name in the line):

Signature of the Participant (Put your signature in the line):

Date:
