# Supplementary material for: Associated Factors of Mycobacterium Leprae Infection among People with Leprosy in Kwale County
Source: PLoS Negl Trop Dis. 2025 Nov 25;19(11):e0012901. doi: 10.1371/journal.pntd.0012901 (PMC12677770; doi:10.1371/journal.pntd.0012901)
Supplement: S4 Text — (DOCX) [file pntd.0012901.s005.docx]

# Study Questionnaire

## Study Title: ‘Factors Associated with New Leprosy Diagnosis in , Kwale County Kenya,2023

Questionnaire number………….................. Date of interview:(dd/mm/yyyy)

# GPS Cordinates………………………….

## Instructions: This questionnaire is to be administered in an environment, which ensures privacy and confidentiality have strictly been adhered

Consent permitted □ Consent denied □

Interviewee category: Self-□ Proxy (Parent/Legal Guardian)

1. Sub County: …………………………………………….............................
2. Link Facility Name……………………………………………………….
3. Ward……………………………………………………………………….
4. Village…………………………………………………………………….

# Part 1 – Demographic and Social information

1. Age (In years)
2. Gender; Male □ Female □
3. Marital status?

Married □ single □ divorced □ widowed □ widower □

1. What is the religion of the Main respondent?

Christian□ Non-practicing□ Muslim□ Hindu□ Others, specify

1. What is the education level of the participant?

Uneducated □ primary level □ Secondary level □ Tertiary level□

1. What is the main income activity/occupation in this household?

Farming□ Livestock keeping□ Business□ Hunting□ Salaried/Employed□ Fishing□ Unemployed□ Others, specify

1. Has the participant ever experienced a food shortage in her lifetime? Yes □ No □

If yes, In a normal day how many meals do you have?

One meal□ two meals □ three meals □ four meals □

1. When in the usual homestead, where have the participants been getting water for drinking/domestic use?

Piped water □ Borehole□ Spring□ Well□ River/lake/dam□ Others, Specify

Type of participant: Case □ Control □

# Part II: Clinical information and treatment (For Cases only)

1. Date of onset of illness :
2. Date of enrollment for treatment----------------------------------------------- (Ask for the hospital appointment card)
3. Date released from treatment----------------------------------------------------(Ask for the hospital appointment card)
4. How long did it take from the first symptom to seeking treatment? (In months/years):
5. Type/Classification of the participant’s disease (Ask for the hospital appointment card to check the treatment regimen if the participant is not sure)

Paucibacillary□ Multibacillary□

1. Did the participant experience/develop any of the following disabilities/deformities before going for treatment in the hospital?

Grade 0; no impairment□ Grade 1; loss of sensation in hand/foot□ Grade 2, visible impairment□ Others, specify …………………

1. Did the participant experience/develop any of the following disabilities/deformities in the course/after treatment completing treatment?

Grade 0; no impairment □

Grade 1; loss of sensation in hand or foot□ Grade 2, visible impairment□

Others,specify …………………

1. Did the participant seek any treatment/advice elsewhere before going to the hospital? Yes □ No□ If not, skip to Question 18.
2. if yes, where did he/she seek treatment/advice before going to the hospital?

Traditional healer □ Local chemist□ Private clinic□ Others (Specify)

# Part III. Risk factors /Exposure History assessment

1. How long have you lived in this area? More than 5 years □ Less than 5 years? □
2. When in the usual homestead, does the participant has any other Household member diagnosed with the disease previously?

Yes□ No□ If No, skip to Qs 22

1. if yes, how long have you stayed /lived with a member of your Household/family? Up to 5 years □ 6+ years□ Other specify………………………
2. When in the usual homestead, has the participant had any contact with someone who has the disease but not from your Household/family diagnosed with the disease?

yes □ No □ if no, skip to QS 24

1. If yes, how long have you been in contact with them?

Up to 5 years □ 6 Years+ □ Others specify………………

1. Does the participant has a BCG scar on the left arm? Yes □ No □
2. Using a MUAC, assess the Body Mass Index (BMI)/Zscore of the participants………..
3. Does/Has the participant been diagnosed with any of the following diseases previously Tuberculosis□ 2.HIV/AIDS□ 3. TB/HIV□ 4. Cancer□

Diabetes/Hypertension □ other specify…………………………

# Part iv: Human Behavioral factors

1. Does the participant usually share own(current) bed linen? Yes□ No□
2. Does the participant usually share other bed linen? Yes □ No□
3. How frequently does the participant change own bed linen?

<Biweekly□ >Bimonthly□ Quarterly□ Biannually□

1. Does the participant has a bathroom? Yes□ No□
2. Between the sunset and the time (s) he goes to sleep, for the previous 5 years, have you ever been bathing in the open (dam, river, lake)

Yes □ No□

1. Are domestic animals available in this home? Yes□ No□ If no, go to Qs. 38
2. Are there usually animals at night in this house yard where (s) he is used to sleeping? (including animals located outside in animal accommodation pens)

Yes□ 2. No□

1. During the last 5 years, did the participant practice hunting in the forest? Yes□ No□
2. During the last 5 years, did the participant practice fishing? Yes□ No□
3. During the last 5 years, did the participants ever live in the following areas as places of usual residence or in search of pastures/relatives?

Kilifi/Kwale/Mombasa□ Bungoma/Busia□ Homabay/Siaya/Kisumu/migori□ Others, specify

# Part v: Household and Environmental factors

1. Between the sunset and the time (s) he goes to sleep, what are the number of rooms in the homestead

<3□ 3-5□ >6□ other, specify………………………………………

1. Between the sunset and the time (s) he goes to sleep, what is the number of your household members, including the participants?

<5□ 5-10□ >10 □ others, specify……...

1. During the last 5 years, did the participant ever worked/works on an agricultural farm? Yes□ No□

# Observation: Ask the participants or the caretaker to show you the room

**where the participant sleeps when they are in the House (if there are several rooms, ask for the main room and as well observe availability and use of the toilets)**

1. What type of floor in the room where they use for sleeping on? observe

Earth □ Earthen □ Cemented/tiled□ A mixture of animal dung□ Others specify…….

1. What is the type of House in the participant’s usual home? oberv

Temporal grass thatched house□ Permanent house□ Manyatta□ Semi-permanent house□ others, specify

1. In the usual homestead, where does the participant usually go for toilet purposes for the previous 5 years? Observe

Personal Latrine/Toilet□ In the Neighbour’s Latrine□ In the bush/open field□ others specify…………………………

# Part VI:General Knowledge and practices of Leprosy in Kwale County

1. Are you aware of the disease? (Before illness for cases) Yes□ No□
2. How can one get leprosy (Do not read)

Airborne□ Contact with infected person□ witchcraft□ inheritance□ Others, specify

1. What are the signs and symptoms of leprosy? (Do not read)

loss of sense in the hands and feet□ Any weakness in hands and feet□ Any wounds on hands and feet Any problems in relationships or participating in festivities, work, or meetings□ Others, List

1. How can someone with leprosy be cured? (Do not read)

Drugs from health facility□ Traditional medicine □ No, cure□ Do not Know□ Others, specify

1. Do you know anyone who got sick, has been treated, hospitalized, on medication, or died because of leprosy?

Yes□ No□

1. Do you think leprosy is a severe problem in your community? Very serious□ Serious□ Not serious□ Do not know□
2. Have you received any information about leprosy in the last 6 Months? Yes□ No□
3. If yes,what was your Sources of the information on leprosy?

Radio/Television□ Health care worker□ Flyers/Brochures□ Chief□ CHV,s□ Others specify

1. Do you practice any of the following leprosy prevention and control measures? (Read Furthermore, allow the participant to name others not listed?)

□Use of bed nets □Use of insect repellants for humans □Use of insect repellants for animals □Indoor spraying with insecticide □Filling cracks on walls □Fighting

rodents □Cutting trees □Killing dogs □ Others specify □ I don’t practice any

1. Would you wish to receive more information on leprosy? Yes□ No□
2. What was the preferred sources of receiving more information about leprosy?

Radio Health care worker Flyers/Brochure Chief Barraza’s Television 7. Megaphone/Public address systems CHV, s Others (Specify)
