# Supplementary material for: Associated Factors of Mycobacterium Leprae Infection among People with Leprosy in Kwale County
Source: PLoS Negl Trop Dis. 2025 Nov 25;19(11):e0012901. doi: 10.1371/journal.pntd.0012901 (PMC12677770; doi:10.1371/journal.pntd.0012901)
Supplement: Attachment — Submitted filename: Response To Reviewers.pdf [file pntd.0012901.s008.pdf]

Rebuttal Letter for Manuscript NO: PNTD-D-25-00190

Factors Associated with New Leprosy Diagnosis in Kwale County, Kenya

Below is a brief overview, a point-by-point response to each reviewer and editors' comment. Reviewer comments are in bold and authors responses follow (Line reference provided). Revisions made in the article are highlighted with tracked changes.

Point by Point Response

Reviewer: Gilbert Batista

1. **General comments**

The observations made on the first manuscript have been considered in this document.
This study identified factors associated with the diagnosis of new cases of leprosy.
The case-control study methodology is justified because the prevalence is very low, and the incubation period of the disease is long.

The study is interesting because the authors evaluated many socio-economic, environmental, and social factors and the study confirm the results already known, namely that family and social contacts of leprosy cases were at greater risk of developing the disease and that BCG vaccination was a protective factor.

2. **Methodology**

Selection of the controls: lines 204 to 214:

Good methodology for selecting controls. However, in villages or neighborhoods, people often live in compounds where there are several households, so why did the authors not choose controls from the same concession as the cases?

To develop leprosy, one must first have been in contact with someone who has the disease. If a control has never been in contact with a person with leprosy, they will not develop the disease.
Therefore, in my opinion, the case and the control can come from the same household because the control must be in the same conditions as the case in terms of exposure factors.

Authors response:

In our study, we deliberately selected controls from neighboring households rather than from the same household or concession to reduce the likelihood that controls had been exposed to the same source of *Mycobacterium leprae* as the cases. Moreover, controls were matched by age (±10 years) and sex to minimize confounding while ensuring that they were representative of the population at risk but without known exposure to a confirmed leprosy case in their immediate household.

3. <u>Results</u>

Line 290 to 293: "The analysis revealed factors associated with new leprosy diagnosis where the odds of being diagnosed with leprosy was four (4) times higher among those **who have previously been exposed to a family contact** (aOR = 4.33; 95% CI: 2.18–8.58). The odds among those **who had prior exposure to a social contact** was associated with a double odds of new leprosy diagnosis than those without a social contact."

<u>Reviewer Comment</u>: **We are not exposed to a person but to a factor or a fact. Therefore, it is inappropriate to say that someone was exposed to a family or social contact. We should simply say** 'The odds of being diagnosed with leprosy was four (4) times higher among people who were family contacts than**….**'
Or we can say " it is the fact of having previously been a family or a social contact is significantly associated with new leprosy diagnosis.

<u>Authors Response:</u>
Line 283-289: The analysis revealed factors associated with new leprosy diagnosis where the odds of being diagnosed with leprosy was seven (7) times higher among individuals with a family size ≥5 members (aOR = 6.99; 95% CI: 2.71–18.06), than those from a family size with <5 members. The odds of being diagnosed with leprosy was four (4) times higher among people who were family contacts (aOR = 4.33; 95% CI: 2.18–8.58). It is the fact of having previously been a social contact (aOR = 2.24; 95% CI: 1.16–4.32) and non -vaccination with BCG (aOR = 2.24; 95% CI: 1.11–4.53) are significantly associated with new leprosy diagnosis.

4. <u>Conclusion:</u>

"Leprosy prevention interventions in high burden Counties in Kenya could prioritize minimizing family or social contact with leprosy cases."

<u>Reviewer Comment:</u> **How can family contact with leprosy cases be reduced? The leprosy case may be the father, mother, son, etc. They live in a community.**

It is the same comment for this sentence "Similarly, addressing overcrowding and food insecurity, which worsen the spread of the disease, is crucial as well". How can we address overcrowding?

<u>Authors response</u>

Line 346-356: Our results conclude that the odds of being diagnosed with new leprosy disease was higher among individuals from households with a family size of ≥5 members than those from a family size < 5 members. The study also concludes that it is fact of having previously been exposed to a family or social contact is significantly associated with new leprosy diagnosis. In addition, non-vaccination with BCG is associated with new leprosy diagnosis. The study findings highlight the role of close family interactions within nuclear families among extended relatives, workmates and friends may sustain the transmission of leprosy in affected communities. Leprosy prevention efforts in high-burden counties should prioritize early detection, prompt treatment of cases, enhanced community-based surveillance and household contact tracing to reduce transmission. In addition, the ministry of Health should sustain and expand the BCG vaccination coverage among all eligible persons.

Reviewers' Comments:

Reviewer #1: - The objectives of the study are clearly stated
- The design of this case-control study is appropriate for the objectives set, as the prevalence is low and the incubation period of the disease is long
- The population is clearly described for both cases and controls
- The sample size is sufficient
- Correct statistical analyses were used to support the conclusions

Reviewer #2: (No Response)

Reviewer #3: No comment

Reviewer #4: Objectives are clear, but the hypothesis should have been articulated more explicitly. The design is appropriate but has potential biases that should be acknowledged more explicitly. The population is well described and mostly appropriate, though definitions of "cases" and reliance on proxy measures should be refined. Adequate for primary objectives but underpowered for less common exposures. Appropriate methods were applied, but reliance on stepwise selection weakens robustness. Ethical standards are met.

Authors response;

Line 331-344: Our study had limitations. Recall bias was a possible concern due to the study design, though the study considered it during questionnaire development and pretesting. Using a BCG scar as a proxy for vaccination status may have led to misclassification as some vaccinated individuals do not develop visible scars. We used stepwise logistic regression to identify significant associations; however, this method may exclude important confounders, rely heavily on statistical significance. Control selection from neighboring households may have introduced overmatching, potentially underestimating associations due to shared environmental or socio-economic factors. Additionally, the analysis did not adjust for clustering effects at the village level, which may have led to underestimated standard errors. Also, unmeasured variables such as genetic susceptibility and undiagnosed subclinical cases could partly explain the observed associations. Despite these limitations, the frequency-matched 1:3 case-control design enhanced statistical power and remains a robust approach for investigating rare diseases like leprosy. Future studies incorporating longitudinal follow-up and broader contextual factors are recommended to validate and expand upon these findings.

Line 140-144: Cases were people identified from the County leprosy treatment registers who had received treatment for leprosy between January,2022 and May, 2023.The individuals were diagnosed based on clinical presentation and where available, supported by laboratory results. In Kenya, leprosy diagnosis mainly relies on clinical signs and symptoms such as skin lesions, sensory loss, nerve thickening and disability, rather than routine slit-skin smears diagnosis [2], [37].

Results:

Reviewer #1: The results are clearly presented, However, there are some sentences that need to be reworded.

Reviewer #2: (No Response)

Reviewer #3: No comment

Reviewer #4: Mostly consistent, but minor discrepancies reduce clarity. Tables are comprehensive but crowded. Some results (e.g., recall bias effect) are mentioned but not quantified. Tables are informative, but: Some percentages do not sum exactly (rounding errors). Formatting could improve readability (e.g., separating crude and adjusted models). No graphical representation (forest plots, bar graphs), which would aid interpretation.

Conclusions:

Reviewer #1: The data supported the conclusions but there are some sentences that need to be reworded.

Reviewer #2: (No Response)

Reviewer #3: No comment

Reviewer #4: Yes. The authors conclude that larger household size, family/social contact, and lack of BCG vaccination are major risk factors for new leprosy diagnosis. These are directly supported by the logistic regression findings. However: The causal language ("predictors") should be tempered, since this is an observational study. Alternative explanations (e.g., genetic susceptibility, subclinical cases) are not addressed. Potential overmatching of controls is not acknowledged. Stepwise regression's limitations are not adequately discussed suggest that findings inform targeted interventions, such as household-level screening and vaccination campaigns. The study contributes to contextualizing global leprosy knowledge into a local Kenyan setting, advancing understanding of socio-economic risk markers. This provides actionable insights for the Kenyan national program and aligns with WHO leprosy strategies.

Authors response;

Causal Language:
We have revised the manuscript to replace terms such as "predictors" or "determinants" with more appropriate phrasing such as "associated factors" and below is a revised version of the study limitations;

Line 346-356: Our results conclude that the odds of being diagnosed with new leprosy disease was higher among individuals from households with a family size of ≥5 members than those from a family size < 5 members. The study also concludes that it is fact of having previously been exposed to a family or social contact is significantly associated with new leprosy diagnosis. In addition, non-vaccination with BCG is associated with new leprosy diagnosis. The study findings highlight the role of close family interactions within nuclear families among extended relatives, workmates and friends may sustain the transmission of leprosy in affected

communities. Leprosy prevention efforts in high-burden counties should prioritize early detection, prompt treatment of cases, enhanced community-based surveillance and household contact tracing to reduce transmission. In addition, the ministry of Health should sustain and expand the BCG vaccination coverage among all eligible persons.

Editorial and Data Presentation Modifications?

Use this section for editorial suggestions as well as relatively minor modifications of existing data that would enhance clarity. If the only modifications needed are minor and/or editorial, you may wish to recommend “Minor Revision” or “Accept”.

Reviewer #1: I recommend "Accept" after minor corrections

Reviewer #2: (No Response)

Reviewer #3: No comment

Reviewer #4: Major revision required

Summary and General Comments:

Reviewer #1:
This study identified factors associated with the diagnosis of new cases of leprosy.
The case-control study methodology is justified because the prevalence is very low, and the incubation period of the disease is long.
The study is interesting because the authors evaluated many socio-economic, environmental, and social factors and the study confirm the results already known, namely that family and social contacts of leprosy cases were at greater risk of developing the disease and that BCG vaccination was a protective factor.

Reviewer #2: Introduction
- Authors should familiarise with the standard binomial convention for naming micro-organisms. This was a comment in the previous round of review and should be taken seriously. The species epithet should be in the lower case.

- Authors should provide estimates of leprosy incidence/prevalence in Kenya in the introduction
-Line 78: Greater caution should be exercised in causally attributing occurrence of disability in leprosy to health system factors.

-Line 83: More clarity is needed in the phrase "consistently reported leprosy cases for the past decade". Does this mean leprosy cases were reported every year for the past decade?
-Line 85: National average should be reported for context.
-Line 86: More caution in causal attributions. Moreover, it is not clear how these ecological factors facilitate transmission. More clarity is needed.
Line 91: "However, these factors may manifest differently in this setting due to the distinct

lifestyle and healthcare barriers" Not sure what this means, or what point is being made here.
Line 96: The genus name/abbreviation should be capitalised
Line 98: Would it be better to say "more susceptible" rather than "at the highest chance"?

Authors response;

Line 74-75: In 2021, the estimated national prevalence was 0.21 cases per 10,000 population [9]

Line 80-82: This may suggest substantial delays in diagnosis, which may arise from a combination of individual factors such as low disease awareness and systemic issues, including limited access to early detection and treatment services

Line 86-89: The county has been reporting leprosy cases every year for the past decade, consistently contributing a significant proportion of the national burden (n=1123). Over this period, it accounted for approximately 24.3% of all reported cases in Kenya, well above the national average [11]. The sustained burden

Line 94-105: Previous studies have identified risk factors such as age, malnutrition, close contact with leprosy patients, and poverty [7], [17], [18]. In this setting, these factors may have an influence to leprosy transmission due to overcrowded living conditions, poor health-seeking behaviors and limited public health infrastructure.

Line 205; exposed to *M. leprae* infection through such contact

Line 98-105: Leprosy remains a complex and poorly understood disease, partly due to its long incubation period—ranging from three to seven years for paucibacillary and up to seventeen years or more for multibacillary forms [7], [19], [20]. People with the multibacillary form of leprosy are considered the main source of M. leprae transmission, primarily through droplets from coughing and sneezing [6], [21]. Those in close contact with untreated multibacillary patients are more susceptible to contracting the disease [22]. Although inhalation remains the most widely accepted transmission route, other possibilities such as skin-to-skin contact or zoonotic transmission, have been suggested by scholars. [23], [24]. Most infected persons remain asymptomatic

Methods
- Line 135-136: Should be revised. Sounds confusing, as though leprosy is the treatment regimen.

- The text mentions cases were "treated with leprosy based on clinical/laboratory confirmation" but then states that Kenya "mainly diagnoses clinically and epidemiological criteria, due to limited slit-skin smear use." This contradiction needs clarification.

- I have read Clinical, laboratory, epidemiological criteria more then twice and still cannot decipher what this criteria is. The authors should be more direct and specific about what point

they are trying to pass. If the cases were selected based on being on treatment and the authors have no access to the diagnostic criteria utilised, then the authors can succinctly mention how the cases were identified for their study.

- Uncertain why the case and control definitions are repeated in the study design section.

Operational definitions: This section is redundant and repeating what has already been said.

Authors Response;

Line 139-153:A 1:3 Case-Control study was conducted between June and September 2023 in rural Kwale County. Cases were people identified from the County leprosy treatment registers who had received treatment for leprosy between January,2022 and May, 2023.The individuals were diagnosed based on clinical presentation and where available, supported by laboratory results. In Kenya, leprosy diagnosis mainly relies on clinical signs and symptoms such as skin lesions, sensory loss, nerve thickening and disability, rather than routine slit-skin smears diagnosis [2], [37].

Controls were individuals without signs or symptoms of leprosy, residing in neighboring households to the cases (from a next or nearby plot), and matched by sex, age group (±10 years) and village. They were assessed for leprosy symptoms using a structured checklist ("S3 C") combining self-report and direct clinical observation by trained research assistants.

Leprosy treatment in Kenya follows WHO guidelines: patients diagnosed with either Paucibacillary or Multibacillary leprosy receive multidrug therapy which includes rifampicin, dapsone, and clofazimine over a 6 to 12-month period, through the National Tuberculosis and Leprosy Programme [1], [37], [38].

Kwale County, located along Kenya's coast and bordering Tanzania, has an estimated population of 820,199, with 51% female and 47.4% living below the national poverty rate at 36.1% [15][14]. The region includes both urban and rural settings, with Ukunda as a central urban centre. Challenges include poor sanitation, limited access to water and a dual burden of diseases such as HIV (2.9% prevalence) and 992 tuberculosis cases diagnosed in 2021[9][39]. Leprosy treatment is available at all the level 4 hospitals in the County. Approximately 73%-75% of the population live within 7 km of these facilities, while 13% live 5–10 km away and about 14% live more than 10 km away

Line 163-170; Case: Any person recorded in the treatment register and treated for leprosy between January 2022 and May 2023.Control: An individual with no clinical signs of leprosy, matched to a case by sex, age group (±10 years), and residence, and residing in a neighboring household. Family contact: contact with an individual who lives in the same household and shares a kitchen with a person diagnosed with leprosy. Social contact: Contact with someone from outside the immediate household but with frequent social interaction or shared kitchen space (e.g., relatives, friends, schoolmates, co-workers). Neighbor: An individual who lives in a separate but nearby household (next or adjacent plot) in the same village.

Reviewer commends: Sample Size, Assumptions and Sampling Procedure

- Line 178-181: This sentence is confusing "Using close contact with known leprosy cases as the exposure of concern, approximately 60% of controls were considered exposed to Mycobacterium Leprae Infection." How was exposure defined? Are the authors saying not all controls were considered exposed?
- The authors state that "approximately 60% of controls were considered exposed to Mycobacterium Leprae Infection". This is a very specific and high estimate for a general population sample. The manuscript provides no source or rationale for this figure.

- "Least extreme odds ratio" is confusing. Do you mean minimum detectable OR?
-Why choose OR of 2.6 as a clinically meaningful benchmark?
- The phrasing suggests this might be a post-hoc power calculation, which is methodologically questionable.
- Mentioning both "significance level of 95%" and "95% confidence interval" is repetitive and unnecessary.
- The case and control selection procedure should come much earlier.

Authurs Response:

Line 170-210; **Selection of the cases**:The County Tuberculosis and Leprosy Coordinator provided the list of all the leprosy treatment facilities. A list of all people who had received treatment for leprosy between January 2022 and May 2023 were abstracted from the leprosy treatment registers ("S1 A"). The lead investigators signed the data confidentiality declaration form ("S2 B") with all the treatment facilities to ensure patient's records were kept confidential and private. The data protection method of de-identification was applied to replace personal characteristics with unique numbers, making it impossible to re-establish a link between his or her data. Both verbal and written consent was sought from adults above 18 years and assent administered to children by seeking permission from the guardians at household level ("S4 D"). Only individuals who gave consent/assent were enrolled in the study. Patients who who mate the criteria were enrolled sequentially until the required sample size was reached. The identified cases were traced to their respective villages and places of residence. The validated questionnaire ("S5 E") was used for interviews at the household level. The scar on the left forearm was to identify individuals who had received the Bacillus Calmette-Guérin (BCG) vaccination.

Selection of the controls

Controls were identified and followed from the exact village as cases. A comprehensive list of all households in the village or residence was obtained from the village elder. Each household was assigned a unique number and simple random sampling technique was used to select a neighboring household to cases using random numbers generated from MS excel tables. In this regard, the first household was selected systematically where, an eligible control matching the case age group category ± 10 years and sex was selected. At home, both written and oral consent/assent was sought to conduct interviews in the households irrespective of the age. If

more than one eligible person was present one was randomly selected using secret balloting method by use of papers marked yes or no. In the event of absence or refusal, the household was dropped, and the next household on the randomized list was selected. This was repeated until each village had the required target of controls. Controls were screened using a structured checklist ("S3 C") to ensure that they had no visible signs or symptoms of leprosy.

Sample size and Assumptions

The Kelsey formula was used to compute the sample size [40]. A total of 65 cases and 195 controls were included in the study. Using close contact with known leprosy cases as the exposure of concern, it was estimated based on previous literature that approximately 60% of controls were exposed to M. leprae infection through such contact[4]. Exposure in this context was defined as either family, social, or neighbor contact with a person diagnosed with leprosy. The minimum detectable odds ratio (OR) was set at 2.6, considered a clinically and epidemiologically meaningful effect size in similar community-based leprosy studies. With this assumption, the sample size was sufficient to detect differences in exposure between cases and controls at 80% power and 95% confidence interval.

Reviewers commend;Why do the authors have study limitations in the methods?

<u>Authors response</u>

Line 331-344: Our study had limitations. Recall bias was a possible concern due to the study design, though the study considered it during questionnaire development and pretesting. Using a BCG scar as a proxy for vaccination status may have led to misclassification as some vaccinated individuals do not develop visible scars. We used stepwise logistic regression to identify significant associations; however, this method may exclude important confounders, rely heavily on statistical significance. Control selection from neighboring households may have introduced overmatching, potentially underestimating associations due to shared environmental or socio-economic factors. Additionally, the analysis did not adjust for clustering effects at the village level, which may have led to underestimated standard errors. Also, unmeasured variables such as genetic susceptibility and undiagnosed subclinical cases could partly explain the observed associations. Despite these limitations, the frequency-matched 1:3 case-control design enhanced statistical power and remains a robust approach for investigating rare diseases like leprosy. Future studies incorporating longitudinal follow-up and broader contextual factors are recommended to validate and expand upon these findings.

Data management and statistical analysis

- Authors have not addressed statistical comments from the previous round of review.

- Maybe this was not clear in the previous round of review, stepwise methods are criticized because they use a purely algorithmic approach to determine model composition, which can lead to the exclusion of important confounders or the inclusion of statistically significant but clinically irrelevant variables.

- For an explanatory study like this, a more robust approach, such as purposeful selection based on prior epidemiological knowledge and assessing changes in estimates, would have been more appropriate.

- The authors state they frequency-matched controls to cases on sex, age-group (±10 years), and village. However, the logistic regression model used for the analysis fails to account for this matching. The matching variables are not included as covariates in the final model presented in Table 4. The analysis must accounts for the matching : either include the matching variables (sex, age) in the model or use a conditional logistic regression.

- Authors should consider clustering effects by village. If not accounted for, acknowledge as limitation

<u>Authors Response:</u>

Line 219-235: Data were analysed using MS Excel and STATA version 16.1. Descriptive analysis summarized continuous variables as means and standard deviations. While categorical variables as proportions. Bivariate logistic regression assessed the associations between independent variables and the leprosy disease. Variables with p-values ≤0.2 were included in the multivariable analysis. Stepwise backward elimination was applied to build the best fitting model, given the multiple variables under consideration and the retrospective nature of the study design where the outcome is already known. This technique allowed for the efficient selection of the statistically significant variables, we acknowledge the known limitations of stepwise methods — particularly the risk of excluding important confounders or including variables that are statistically but not clinically relevant Factors with p-values ≤0.05 were retained in the final model. Adjusted odds ratios with 95% confidence intervals were reported to determine the significance of associations [41]. Although cases and controls were frequency-matched on sex, age group (±10 years), and village, the final model did not include these matching variables. We acknowledge this as a limitation in our analysis, as failure to adjust for matching variables may introduce residual confounding. Furthermore, while village-level clustering could potentially affect variance estimates due to intra-cluster correlation, the sample size and distribution of cases across villages limited our ability to adjust for clustering effects statistically.

Results

The presentation of results in the text often merely restates percentages directly from the tables without providing a clear narrative or highlighting the most salient findings.

Discussion

- A flaw in the discussion is the repeated interpretation of non-significant findings as if they were conclusive. For example, the authors state that "persons who skipped meals...were two times more likely to develop leprosy" (Lines 343-345) and that "a low frequency of changing bed linen had higher odds of new leprosy diagnosis" (Lines 350-351). According to the authors' own analysis in Table 4, both of these variables were found to be not significant in the multivariable model. The discussion should be limited to the significant factors identified in the final adjusted model.

- A weakness of the study is the handling of temporality. By selecting participants based on their disease status and then retrospectively assessing exposures, the study cannot definitively establish that the identified "risk factors" preceded the onset of leprosy.
- Because the temporal relationship cannot be confirmed, the authors should be far more cautious in their language. They should frame their findings as associations rather than predictors or causal factors.
- The study notes that a higher proportion of cases were unemployed and experienced food shortages. The authors interpret these as risk factors related to poverty that may increase susceptibility to leprosy. However, it is equally, if not more, plausible that the debilitating physical disabilities and social stigma resulting from leprosy led to job loss and subsequent poverty, thereby causing food insecurity. In this scenario, unemployment and hunger are likely consequences, not causes, of the disease.
- A large family size is a plausible risk factor for increased transmission, however, other household factors could also be subject to reverse causality. The economic impact of leprosy on a household could lead to a deterioration in living conditions, such as the inability to maintain a home

- The link between the study's stated aims and its limitations needs to be better reconciled. The authors state they aimed to evaluate "socio-cultural" factors but then list not investigating "an elaborate social-cultural attitude" as a limitation. This suggests a disconnect between the intended scope and the actual execution of the research, which should be more directly acknowledged.

<u>Authors Response</u>:

Line 293-235; In this study, individuals from large family size, having previously been exposed to a family or a social contact with leprosy and those with non-vaccination with BCG were found to be significantly associated with new leprosy diagnosis. These associations highlight the need for targeted public health interventions such as timely household contact tracing, door-to-door screenings, and sustained or expanded BCG vaccination programs for all eligible populations. Our findings revealed a clear difference between case and control households' sizes where people from households with five or more members had higher odds of being diagnosed with leprosy compared to those from smaller households. This was consistent with previous research in Ethiopia, which highlighted the epidemiological relationship between overcrowding and vulnerability to new leprosy diagnosis [27], [36],], possibly due to increased contact time between infected and susceptible individuals [42]. However, findings by Wagner et al, (2015) in India, showed no association of large family size members with support of leprosy development [33].

We also found that having a known family contact with leprosy was significantly associated with leprosy diagnosis. Our findings were in agreement with previous studies from Ethiopia, Brazil, and Ghana that demonstrate the role of close and prolonged contact in facilitating M. leprae transmission, particularly in cases of Multibacillary leprosy [4], [18], [27]

Our study found that exposure to social contacts (non-household) with leprosy was also significantly associated with new leprosy diagnosis. This is consistent with findings from a

study done in Ethiopia by Bekala et al,2021, that tend to confirm that social contacts as a potential risk factor for leprosy disease [34] [27] [19]. In contrast, Ofusu et al, (2011) did not find a significant connection between social contacts and leprosy, possibly due to differences in control selection [4]. Our findings suggest that leprosy contact investigations could be expanded to include not only household members but also extended relatives, friends, co-workers, and classmates. Delayed detection in such contacts, as shown in other studies in Ethiopia by Urgesa et al (2021)[43], may sustain transmission, especially in endemic areas [8].

Similarly, our study revealed that non-vaccination with Bacillus Calmette Guerin (BCG), as indicated by the absence of a BCG scar on the left forearm, was associated with new leprosy diagnosis. Our findings revealed that a mong the controls, only 16.9% had no BCG scar, compared to 43.1% of the cases. Our results are supported by studies conducted from Brazil, which show a protective effect of BCG vaccination against new leprosy diagnosis. Although our data indicate a strong association between non-vaccination and leprosy disease, further research is required to better understand the extend and duration of protection partly conferred by BCG vaccination to eligible populations.[4], [5].

Conclusion

The conclusions and author summary should align precisely with the final, statistically significant results. The mention of "food insecurity" in the author's summary (Line 59) is not supported by the final multivariable analysis and should be removed to avoid misrepresenting the study's findings.

<u>Reviewer commends #3</u>: This version looks good to me

Reviewer #4:

Strengths: Timely and context-specific, addressing leprosy in a high-burden Kenyan county. Clear case-control methodology with robust sample size. Strong ethical oversight and community engagement. Identification of actionable predictors (household size, BCG, contact patterns). Results have immediate programmatic relevance for leprosy control.

Weaknesses: Hypothesis not explicitly stated in testable terms. Stepwise regression risks overfitting and weakens interpretability. Control selection may bias results (neighbors share exposures).Over-reliance on BCG scar as proxy.
Presentation of tables could be clearer; absence of figures weakens impact.
Some causal language is overstated for observational data

<u>Authors response;</u>

Line 346-356; Our results conclude that the odds of being diagnosed with new leprosy disease was higher among individuals from households with a family size of ≥5 members than those from a family size < 5 members. The study also concludes that it is fact of having previously been exposed to a family or social contact is significantly associated with new leprosy diagnosis. In addition, non-vaccination with BCG is associated with new leprosy diagnosis. The study

findings highlight the role of close family interactions within nuclear families among extended relatives, workmates and friends may sustain the transmission of leprosy in affected communities. Leprosy prevention efforts in high-burden counties should prioritize early detection, prompt treatment of cases, enhanced community-based surveillance and household contact tracing to reduce transmission. In addition, the ministry of Health should sustain and expand the BCG vaccination coverage among all eligible persons.
