# Supplementary material for: Associated Factors of Mycobacterium Leprae Infection among People with Leprosy in Kwale County
Source: PLoS Negl Trop Dis. 2025 Nov 25;19(11):e0012901. doi: 10.1371/journal.pntd.0012901 (PMC12677770; doi:10.1371/journal.pntd.0012901)
Supplement: Attachment — Submitted filename: Response To Reviewers.pdf [file pntd.0012901.s010.pdf]

Rebuttal Letter for Manuscript NO: PNTD-D-25-00190

Factors Associated with New Leprosy Diagnosis in Kwale County, Kenya

Below is a brief overview, a point-by-point response to each reviewer and editors' comment. Reviewer comments are in bold and authors responses follow (Line reference provided). Revisions made in the article are highlighted with tracked changes.

Point by Point Response

Reviewer: Gilbert Batista

1 Title:
Reviewer Comment:
It is preferable to speak of factors associated with leprosy rather than factors associated with infection with *Mycobacterium leprae*, because the notion of infection makes us think of asymptomatic cases with documented latent infection.

Authors response: Factors Associated with New Leprosy Diagnosis in Kwale County, Kenya (Line 1).

2) Abstract
Background
Despite maintaining the global elimination target of <1/10000 population, Kenya reported a six-fold increase in cases from 2011 to 2021, with Kwale County contributed 24.3%.
Reviewer Comment:
Enter the number of leprosy cases reported in 2011 and 2021

Authors response: Reported a six-fold increase in cases (26-163) from 2011 to 2021, with Kwale County contributed 24.3% (Line 24).

3) Line 133: Controls were people with no signs and symptoms and were a neighbor to a case;
Reviewer Comment:
Describe who the neighbor is; is it a member of the same household, a neighboring household in the same plot or another plot? It is known that the probability of contracting leprosy increases the closer you are to the index case.

Authors response: Controls were people with no signs and symptoms of leprosy and were from a neighboring household to a case from another, next or nearby plot, matched to a case by sex, age group of ±10 years and village, residence or community (Lines 140-142).

4) <u>General comments</u>

- **An interesting study which confirms that family and social contacts are more likely to develop leprosy in a context of poverty and promiscuity.**
  Authors response: Thank you

- **The selection of controls was well done, but even if they are in the same village, they must not be far from the cases.**

  Authors Response: Neighbor to a case from another, next or a nearby plot within the same village, residence or community.

- **<u>Reviewer comment</u>-In a case-control study like this one, we look for previous exposure factors that are associated with the disease. It will be necessary to explain that it is the fact of having previously been a family contact or a social contact that is significantly associated with leprosy. Similarly, it should be said that non-vaccination with BCG is associated with leprosy and not the absence of a BCG scar because the previous exposure was the vaccination which is evidenced by the scar.**

  Authors response: Individuals from large family size ≤5 members, having previously been exposed to a family or a social contact is significantly associated with leprosy. Similarly, non-vaccination with BCG is associated with leprosy.

Methods

<u>Reviewer #1</u>:

The objectives are clear;

Good methodology: The selection of controls was well done. It's a good idea to choose cases, patients who have just finished treatment or who are currently undergoing treatment

However, describe more who the control is; is it a member of the same household, a neighboring household in the same plot or another plot?

Authors Response: Controls were people with no signs and symptoms of leprosy and were from a neighboring household to a case from another, next or nearby plot, matched to a case by sex, age group of ±10 years and village, residence or community (Lines 140-142).

<u>Reviewer #2</u>:

The method described for selecting control households using a "pen spin" is arbitrary, non-standard, and introduces significant potential for selection bias. This undermines the internal validity of any results.

Authors response: Controls were identified and followed from the exact village as cases. A comprehensive list of all households in the village or residence were obtained from the village elder. Each household was assigned a unique number, and simple random sampling technique

was used to select a neghboring households to cases using random numbers generated from MS excel tables. In this regard, the first household was selected systematically where, an eligible control matching the case sex, age group category ± 10 years was selected. At home, both written and oral consent/assent was sought to conduct interviews in the households irrespective of the age. If more than one eligible person was present, one was randomly selected using secret balloting method by use of papers marked yes or no. In the event of absence or refusal, the household was dropped, and the next household on the randomized list is selected. This was repeated until each village had the required target of controls. (Lines 203-213).

Reviewer comment:

Definition of cases is confusing. It is not clear what the diagnostic criteria is. Is it clinical or laboratory confirmed or both? What time frame did the authors use for "recently completed treatment"?

Authors response: Cases included people who were treated with leprosy based on the clinical/ laboratory confirmation and epidemiological criteria between January 2022 and May 2023, and were identified from treatment registers (Line 138-140).

Reviewer comment:

The method for confirming controls had "no signs or symptoms" is not specified. Was this based on self-report or clinical examination?

Authors response: Controls were people with no signs and symptoms of leprosy and were a neighboring household to a case from another, next or nearby plot, matched to a case by sex, age group of ±10 years and village, residence or community. The controls were evaluated using structured checklist ("S3 C"), based on self-report and clinical observation by the research assistant denoting the signs and symptoms of leprosy (Lines 140-142).

Reviewer Comment:

Key methodological details are missing or unclear, such as the specific age-matching criteria, validation/pre-testing of the questionnaire, and handling of refusals during recruitment.

Authors response: Controls were people with no signs and symptoms of leprosy and were from a neighboring household to a case from another, next or nearby plot, matched to a case sex, age group of ±10 years and village, residence or community (Lines 162-166).

Authors response:A validated questionnaire in a neighbouring County was developed and used to collect data (Lines 216-217).

Authors response: In the event of absence or refusal, the household was dropped, and the next household on the randomized list was selected. This was repeated until each village had the required target of controls (Lines 211-213).

Reviewer comment:

The reliance on a BCG scar as a proxy for vaccination without sufficient acknowledgment of its limitations in the methods section is problematic.

Authors response: The BCG scar on the left forearm was used as a proxy for immunity established from BCG vaccination. it introduced bias since we were not able to distinguish those who got vaccinated but did not develop the scar from those who never got the vaccine. (Lines 223-226).

Reviewer comment:

The statistical analyses subsection is badly written. The rationale for using stepwise backward elimination for model building is not provided, and potential issues with this method are not acknowledged. Why was this method even considered if this was not a prediction study?

Authors response: Data were analyzed using MS Excel and STATA version 16.1. Descriptive analysis summarized continuous variables as means and standard deviations. Categorical variables as proportions. Bivariate logistic regression assessed the associations between independent variables and the leprosy disease. Variables with p-values ≤0.2 were included in the multivariable analysis. Stepwise backward elimination was applied to build the best fitting model, given the multiple variables under consideration and the nature of the study design where the outcome is already known. This technique allowed the efficient selection of the statistically significant risk factors while controlling for potential confounders. Factors with p-values ≤0.05 were retained in the final model. Adjusted odds ratios with 95% confidence intervals were reported to determine the significance of associations (Lines 232-242).

Reviewer #3:

Are the objectives of the study clearly articulated with a clear testable hypothesis stated?

Reviewer comment: An elaborate understanding of the specific socioeconomic factors like low education attainment and low income for leprosy transmission is critical. This seems interesting but will be a challenge to determine the predictors associated with m. Leprae infection in the study setting as there so many determinant interconnected.

Authors response: We found out that persons who skipped meals to deal with hunger often ate one meal (46%) or even slept hungry, were two times more likely to develop leprosy disease (Line 347-348). The skipping of meals was used as a proxy for low income.

Authors response: In addition, we did not investigate on stigma and discrimination experience as well as an elaborate socio-economic, cultural aspects and attitudes for the study participants (Lines 227-229).

Reviewer comment:

Is the population clearly described and appropriate for the hypothesis being tested?

Reviewer comment: "Cases were identified from treatment registers and comprised leprosy patients who recently completed treatment". Need to define more clearly patients who recently completed treatment. What does it mean recently?

Authors response: Cases included people who were treated with leprosy based on clinical/ laboratory confirmation and epidemiological criteria between January 2022 and May 2023, and were identified from treatment registers (Lines 138-140).

Reviewer comment:

Poverty line. Provide figures and sources for the country/location

Response: The county has a population of approximately 820,199, and 51% being female with nearly half living below poverty index at 47.4% compared to the national rate of 36.1% [15][14] (Lines 150-151).

Reviewer comment:

Please provide % of population living <5k; 5-10km; >10km from health facilities providing leprosy treatment of the study area

Authors response: Approximately, between 73%-75% of the County population live within an average of 7 km to the facilities,13% reside within 5 to 10km away and about 14% live over 10 km distance from the leprosy treatment hospitals [15], [39] (Lines 156-159).

Results

Reviewer #1:

In a case-control study like this one, we look for previous exposure factors that are associated with the disease. It will be necessary to explain that it is the fact of having previously been a family contact or a social contact that is significantly associated with leprosy. Similarly, it should be said that non-vaccination with BCG is associated with leprosy and not the absence of a BCG scar because the previous exposure was the vaccination which is evidenced by the scar.

Authors response: The odds of being diagnosed with leprosy was seven times higher among patients with a family size ≥5 members (aOR=6.99, 95% CI: 2.71–18.06) than those from a family size of <5 members and four times higher among those with a family contact (aOR=4.33, 95% CI: 2.18–8.58). Social contact (aOR=2.24,95% CI 1.16–4.32) and non-vaccination with BCG (aOR=2.24,95% CI: 1.11–4.53) is associated with a double odds of new leprosy diagnosis than those without social contact and BCG vaccination (Lines 39-44).

Reviewer #2:

There are numerous inconsistencies between the text descriptions and the data presented in the tables.

- Percentages in tables do not always appear logical or sum correctly. In Table 2, under the "Employment" variable, if you sum these percentages for the Controls: 60.0% + 2.7% + 37.9% = 100.6

Authors response: Employment variable, Controls: 60.0% + 2.7% + 37.3% = 100% (Line 276).

Reviewer #3:

Does the analysis presented match the analysis plan? Yes

Reviewer: Are the results clearly and completely presented? Yes, but I suggest if possible that factors associated to geographic and financial access to health care are taken into account.

Authors response: An elaborate and detailed analysis for geographic and financial status was minimal since they were not significant at the multivariable level

Conclusions

Reviewer #1:

The limitations of analysis are not clearly described

This is an interesting study in a particular context in Kenya which confirms that family and social contacts are more likely to develop leprosy in a context of poverty and promiscuity

Authors response: The study limitations include the selection bias where the controls were selected using a structured screening checklist denoting signs of leprosy. The BCG scar on the left forearm was used as a proxy for immunity established from BCG vaccination. It introduced bias since we were not able to distinguish those who got vaccinated but did not develop the scar from those who never got the vaccine. Another limitation of the study was recall bias among the participants due to the study design, we considered recall when developing and validating the questionnaire. In addition, we did not investigate on stigma and discrimination experience as well as an elaborate socio-economic, cultural aspects and attitudes for the cases and controls (Lines 222-229).

Reviewer #2:

There are flaws in the study design, methodology, data presentation, and interpretation. The conclusions drawn are not adequately supported by the methodology and data presented and the manuscript, in its current form, does not meet the scientific rigor expected for publication.

Authors response: A 1:3 case-control study was conducted in rural Kwale County. Cases included people who were treated with leprosy based on clinical/laboratory confirmation and epidemiological criteria between January 2022 and May 2023, and were identified from treatment registers. Controls were people with no signs and symptoms of leprosy and were from a neighboring household to a case from another, next or nearby plot, matched to a case by sex, age group of ±10 years and village, residence or community (Lines 138-142).

Authors response: Employment" variable, Controls: 60.0% + 2.7% + 37.3% = 100% (Line 276).

Authors response: The odds of being diagnosed with leprosy was seven times higher among patients with a family size ≥5 members (aOR=6.99, 95% CI: 2.71–18.06) than those from a

family size of <5 members and four times higher among those with a family contact (aOR=4.33, 95% CI: 2.18–8.58). Social contact (aOR=2.24,95% CI 1.16–4.32) and non-vaccination with BCG (aOR=2.24,95% CI: 1.11–4.53) is associated with double odds of new leprosy diagnosis than those without social contact and BCG vaccination (Lines 39-44).

Authors response: Our results conclude that the odds of being diagnosed with new leprosy disease was higher a mong patients with a family size ≥5 members than those from a family size < 5 members. The study concluded that it is fact of having previously been exposed to a family or a social contact is significantly associated with new leprosy diagnosis. Similarly, the study concludes that non-vaccination with BCG is associated with leprosy. The study highlights that family or social interactions within nuclear families, distant relatives, workmates and friends may sustain the transmission of new leprosy diagnosis. Leprosy prevention interventions in high burden Counties in Kenya could prioritize to minimizing family or social contact with leprosy cases, enhance community household contact surveillance and household contact tracing especially for larger families. In addition, the ministry of Health should sustain and expand the BCG vaccination coverage among all eligible persons (Lines 377-388).

<u>Reviewer #3</u>:

Are the conclusions supported by the data presented?

Yes. But in summary, all the results were not presented. It focused only result associate to BCG. Please elaborate.

Response: Our results conclude that the odds of being diagnosed with leprosy disease was higher among patients with a family size ≥5 members than those from a family size < 5 members. The study concluded that it is fact of having previously been exposed to a family or a social contact is significantly associated with new leprosy diagnosis. Similarly, the study concludes that non-vaccination with BCG is associated with new leprosy diagnosis. The study highlights that family or social interactions within nuclear families, distant relatives, workmates and friends may sustain the transmission of new leprosy diagnosis. Leprosy prevention interventions in high burden Counties in Kenya could prioritize to minimizing family or social contact with leprosy cases, enhance community-based surveillance and household contact tracing. In addition, the ministry of Health should sustain and expand the BCG vaccination coverage among all eligible persons (Lines 377-388).

<u>Reviewer comment</u>:

Are the limitations of analysis clearly described?

Yes

<u>Reviewer comment</u>:

Do the authors discuss how these data can be helpful to advance our understanding of the topic under study?

Yes, but we need to add the determinants related to geographic and financial access to health services.

Reviewers comments:

Is public health relevance addressed?

Yes, it contributes if the recommendation are more elaborated structuring by Individual, community, environment, health system responsibilities to addressing the transmission of m. Leprae.

Authors response: Leprosy prevention interventions in high burden Counties in Kenya could prioritize to minimizing family or social contact with leprosy cases, enhance community-based surveillance and household contact tracing. In addition, the ministry of Health should sustain and expand the BCG vaccination coverage among all eligible persons. (Lines 384-388).

Reviewer comments:

Could you please be specific in defining the term "recently"

Authors response: Cases included people who were treated for leprosy based on clinical /laboratory confirmation and epidemiological criteria between January 2022 and June 2023 (Lines 28-29).

Reviewers Comments:

Please be specific while defining the term neighbor to a case

Authors response: -Any individual who resides in a different household but in close physical proximity to a case household such as in the next plot or a nearby plot within the same village, residence or community (Lines 175-177).

Reviewer comments:

Does this relate to the people living in the same house?

Authors response: Yes

Reviewer comments:

Would this one only comment address all factors incriminated?

Authors comments: Leprosy prevention interventions in high burden Counties in Kenya could prioritize to minimizing family or social contact with leprosy cases, enhance community-based surveillance and household contact tracing. In addition, the ministry of Health should sustain and expand the BCG vaccination coverage among all eligible persons. (Lines 384-388).

Reviewers comments:

Could you provide more details on the contents of the community interventions?

Authors response: Our study highlighted the need for targeted interventions such as Strengthening community-based surveillance efforts, household contact tracing and door-to-

door screenings, are extremely important in the fight against leprosy for persons who might have prior exposure to a family or social contact (Lines 59-62).

Reviewers comments:

Please provide % of population living <5km,5-10km and >10km from Health facilities providing leprosy treatment?

Authors response: Kwale has five level 4 hospitals, all providing Leprosy treatment services [16]. Approximately, between 73%-75% of the County population live within an average of 7 km to the facilities,13% reside within 5 to 10km away and about 14% live over 10 km distance from the leprosy treatment hospitals [15], [39] (Lines 155-159).

Additional Editor Comments:

As suggested by the reviewers, it is necessary to provide a clearer description of the control group used in this study.

Authors response: Controls were people with no signs and symptoms of leprosy and were from a neighboring household to a case from another, next or nearby plot, matched to a case by sex, age group of ±10 years and village, residence or community (Lines 140-142).

Yours sincerely,

Vallerian Karani
Kenya Field Epidemiology and Laboratory Training Program, Nairobi, Kenya
Email address: vallerian08@gmail.com
Date:2nd July,2025
On behalf of all Co-authors
