# Supplementary material for: Associated Factors of Mycobacterium Leprae Infection among People with Leprosy in Kwale County
Source: PLoS Negl Trop Dis. 2025 Nov 25;19(11):e0012901. doi: 10.1371/journal.pntd.0012901 (PMC12677770; doi:10.1371/journal.pntd.0012901)
Supplement: Attachment — Submitted filename: Reviewer_Batista Gilbert.docx [file pntd.0012901.s006.docx]

**14/04/2025**

**REVIEWER: GILBERT BATISTA**

**Manuscript N° PNTD-D-25-00190**

**Associated Factors of Mycobacterium Leprae Infection among People with Leprosy in Kwale County, Kenya**

1) Title:

**Comment: It is preferable to speak of factors associated with leprosy rather than factors associated with infection with *Mycobacterium leprae*, because the notion of infection makes us think of asymptomatic cases with documented latent infection.**

2) Abstract

Background

Despite maintaining the global elimination target of <1/10000 population, Kenya reported a six-fold increase in cases from 2011 to 2021, with Kwale County contributed 24.3%.

**Comment: Enter the number of leprosy cases reported in 2011 and 2021**

3) Line 133: Controls were people with no signs and symptoms and were a neighbor to a case;

**Comment: Describe who the neighbor is; is it a member of the same household, a neighboring household in the same plot or another plot? It is known that the probability of contracting leprosy increases the closer you are to the index case.**

**4) General comments**

- An interesting study which confirms that family and social contacts are more likely to develop leprosy in a context of poverty and promiscuity.
- The selection of controls was well done, but even if they are in the same village, they must not be far from the cases.
- It's a good idea to choose cases, patients who have just finished treatment or who are currently undergoing treatment.
- In a case-control study like this one, we look for previous exposure factors that are associated with the disease. It will be necessary to explain that it is the fact of having previously been a family contact or a social contact that is significantly associated with leprosy. Similarly, it should be said that non-vaccination with BCG is associated with leprosy and not the absence of a BCG scar because the previous exposure was the vaccination which is evidenced by the scar.
