# Supplementary material for: Associated Factors of Mycobacterium Leprae Infection among People with Leprosy in Kwale County
Source: PLoS Negl Trop Dis. 2025 Nov 25;19(11):e0012901. doi: 10.1371/journal.pntd.0012901 (PMC12677770; doi:10.1371/journal.pntd.0012901)
Supplement: Attachment — Submitted filename: Reviewer_Batista Gilbert 19082025.docx [file pntd.0012901.s009.docx]

**19/08/2025**

**REVIEWER: GILBERT BATISTA**

**Manuscript N° PNTD-D-25-00190R1**

**Factors Associated with New Leprosy Diagnosis in Kwale County, Kenya**

**1) General comments**

The observations made on the first manuscript have been considered in this document.

This study identified factors associated with the diagnosis of new cases of leprosy.

The case-control study methodology is justified because the prevalence is very low, and the incubation period of the disease is long.

The study is interesting because the authors evaluated many socio-economic, environmental, and social factors and the study confirm the results already known, namely that family and social contacts of leprosy cases were at greater risk of developing the disease and that BCG vaccination was a protective factor.

**2) Methodology**

- Selection of the controls: lines 204 to 214:

Good methodology for selecting controls. However, in villages or neighborhoods, people often live in compounds where there are several households, so why did the authors not choose controls from the same concession as the cases?

To develop leprosy, one must first have been in contact with someone who has the disease. If a control has never been in contact with a person with leprosy, they will not develop the disease.

Therefore, in my opinion, the case and the control can come from the same household because the control must be in the same conditions as the case in terms of exposure factors.

**3) Results**

- Line 290 to 293: “The analysis revealed factors associated with new leprosy diagnosis where the odds of being diagnosed with leprosy was four (4) times higher among those **who have previously been exposed to a family contact** (aOR = 4.33; 95% CI: 2.18–8.58). The odds among those **who had prior exposure to a social contact** was associated with a double odds of new leprosy diagnosis than those without a social contact.”

**Comment: We are not exposed to a person but to a factor or a fact. Therefore, it is inappropriate to say that someone was exposed to a family or social contact. We should simply say ‘the odds of being diagnosed with leprosy was four (4) times higher among people who were family contacts than….’**

**Or we can say “it is the fact of having previously been a family or a social contact is significantly associated with new leprosy diagnosis”**

**4) Conclusion:**

“Leprosy prevention interventions in high burden Counties in Kenya could prioritize minimizing family or social contact with leprosy cases.”

**Comment: How can family contact with leprosy cases be reduced? The leprosy case may be the father, mother, son, etc. They live in a community.**

**It is the same comment for this sentence “**Similarly, addressing overcrowding and food insecurity, which worsen the spread of the disease, is crucial as well”. How can we address overcrowding?
